# Supplementary material for: APEC: an accesson-based method for single-cell chromatin accessibility analysis
Source: Genome Biol. 2020 May 12;21:116. doi: 10.1186/s13059-020-02034-y (PMC7218568; doi:10.1186/s13059-020-02034-y)
Supplement: Supplementary file 1 — Additional file 1: Figure S1. The 3 subtypes of CMP cells in the tSNE maps generated by other methods. Figure S2. Clustering performance of the dimension-transformed matrices generated by different algorithms. (a) The tSNE diagrams of the cells from AML patients and three distinct cell lines (LMPP, monocyte and HL60). Different algorithms provided different dimension-transformed matrices for tSNE analysis, i.e., APEC: accesson matrix; cisTopic: topic matrix; LSI: LSI matrix; chromVAR: bias corrected deviation matrix; Cicero: aggregated model matrix. The table below the diagrams contains the average ARI of the cell clustering results for each algorithm. (b) The tSNE diagrams and ARI table for the leukemic stem cells (LSCs) and blast cells from 2 different AML patients only, as in (a). (c) Box-plots showing the ARI values for the clustering of the blast and LSC cells from two AML patients. We sampled different tunable parameters for different algorithms. APEC: the accesson number; cisTopic: the random seed; SnapATAC: the number of principal components and the number of nearest neighbors; LSI: the number of top SVD components; Cicero: the peak aggregation distance; chromVAR: no sampling. Z-score and probability denote different methods of normalizing the dimension-transformed matrices. Center line, median; box limits, upper and lower quartiles; whiskers, 1.5x interquartile range; points, outliers. (d) The average ARI values calculated by down-sampling 50 times from the raw data of the AML cells and three cell lines for each method. The X-axis represents the percentage of down-sampled sequencing reads. Shaded error band: 95% confidence interval. (e) The average ARI values of the noised data sampled from the fragment count matrix of the same dataset used in (d). The X-axis represents the percentage of noised elements in the matrix. Shaded error bar: 95% confidence interval. Figure S3. Super-enhancers predicted by APEC for the scATAC-seq data of cells from AML patients. ( [file 13059_2020_2034_MOESM1_ESM.docx]

Additional File 1: Supplementary Figures for “APEC: an accesson-based method for single-cell chromatin accessibility analysis”

**SUPPLEMENTARY FIGURES**


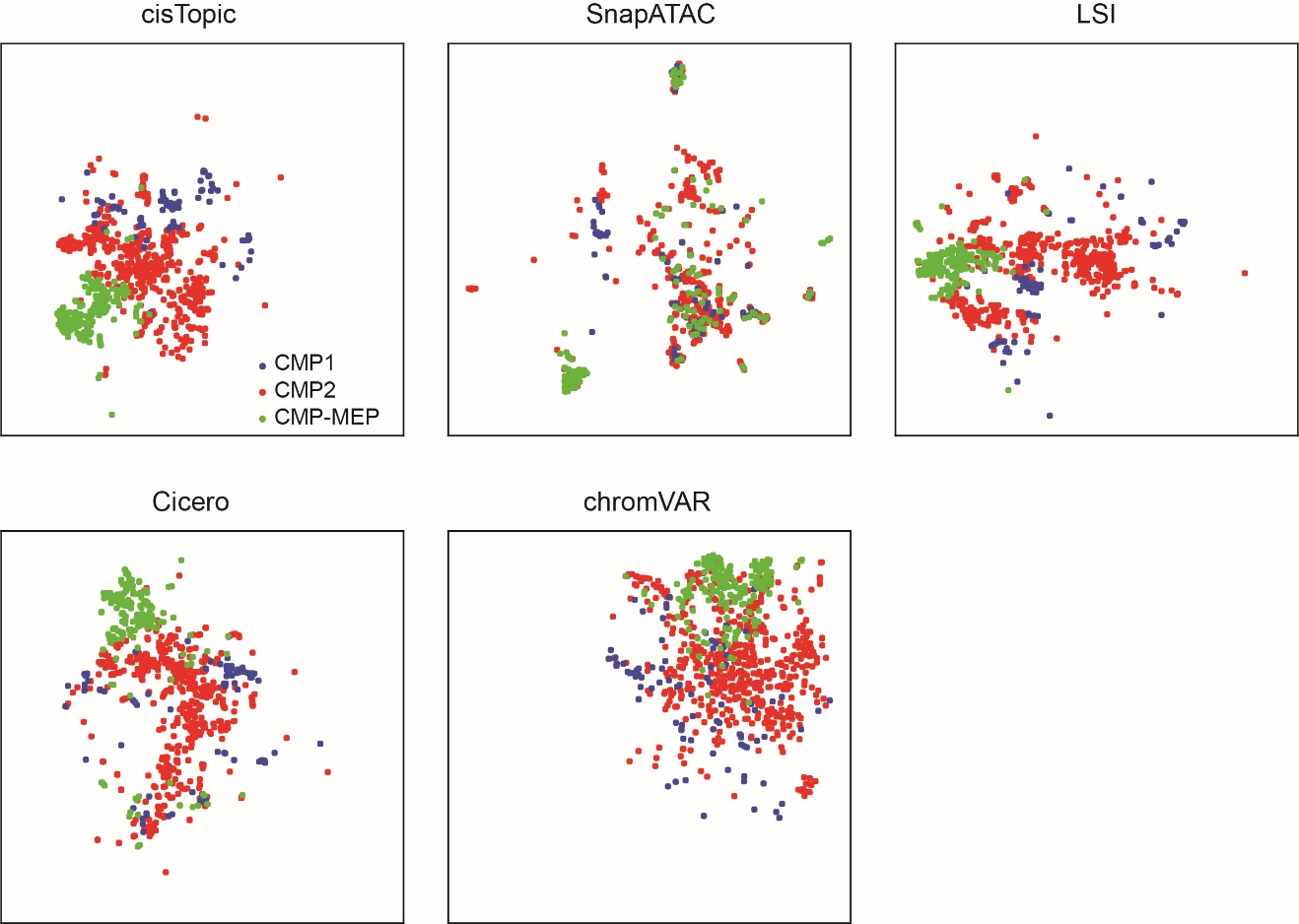


**Figure S1.** The 3 subtypes of CMP cells in the tSNE maps generated by other methods.

**
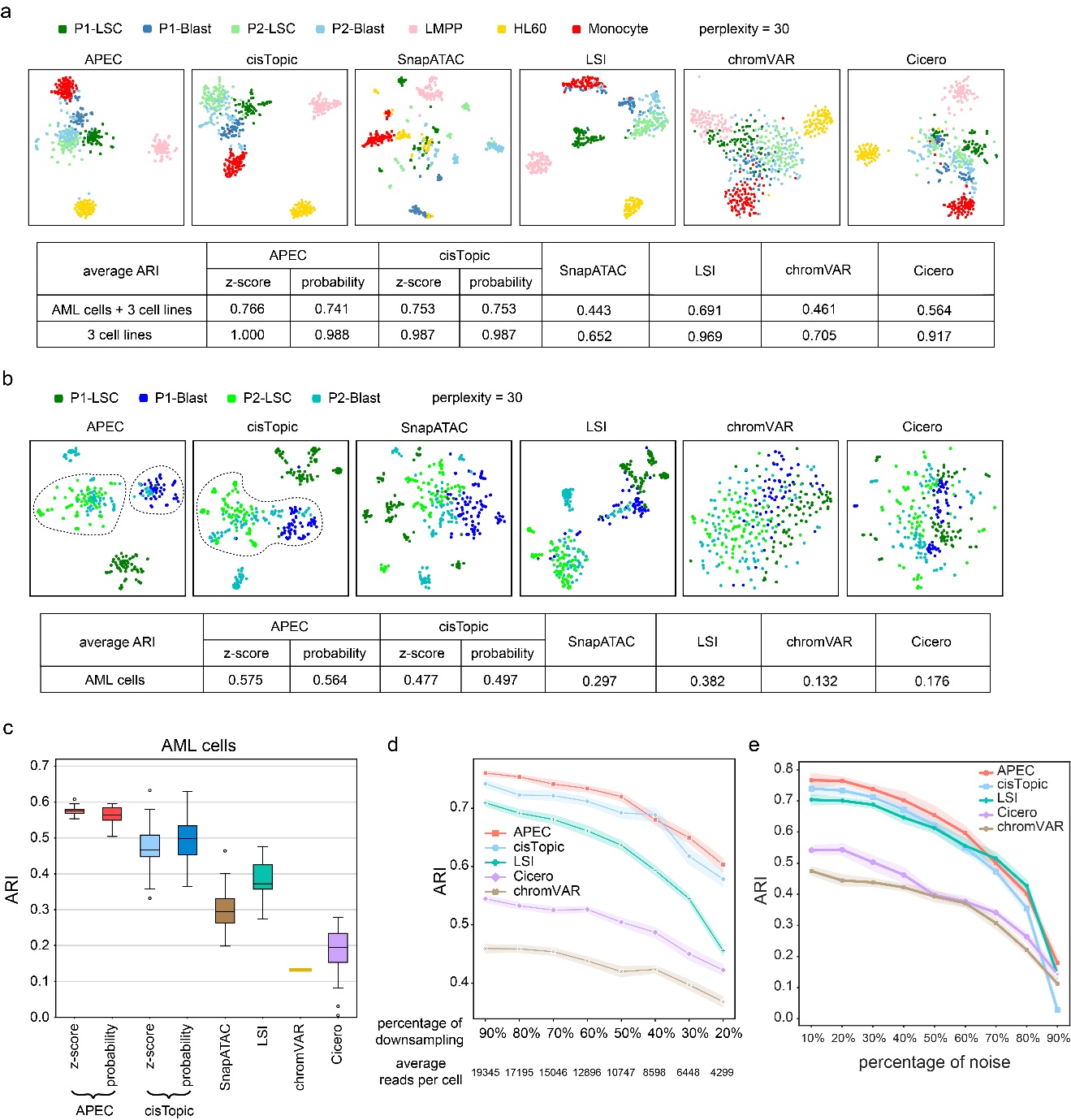
**

**Figure S2.** Clustering performance of the dimension-transformed matrices generated by different algorithms. (**a**) The tSNE diagrams of the cells from AML patients and three distinct cell lines (LMPP, monocyte and HL60). Different algorithms provided different dimension-transformed matrices for tSNE analysis, i.e., APEC: accesson matrix; cisTopic: topic matrix; LSI: LSI matrix; chromVAR: bias corrected deviation matrix; Cicero: aggregated model matrix. The table below the diagrams contains the average ARI of the cell clustering results for each algorithm. (**b**) The tSNE diagrams and ARI table for the leukemic stem cells (LSCs) and blast cells from 2 different AML patients only, as in (a). (**c**) Box-plots showing the ARI values for the clustering of the blast and LSC cells from two AML patients. We sampled different tunable parameters for different algorithms. APEC: the accesson number; cisTopic: the random seed; SnapATAC: the number of principal components and the number of nearest neighbors; LSI: the number of top SVD components; Cicero: the peak aggregation distance; chromVAR: no sampling. Z-score and probability denote different methods of normalizing the dimension-transformed matrices. Center line, median; box limits, upper and lower quartiles; whiskers, 1.5x interquartile range; points, outliers. (**d**) The average ARI values calculated by down-sampling 50 times from the raw data of the AML cells and three cell lines for each method. The X-axis represents the percentage of down-sampled sequencing reads. Shaded error band: 95% confidence interval. (**e**) The average ARI values of the noised data sampled from the fragment count matrix of the same dataset used in (d). The X-axis represents the percentage of noised elements in the matrix. Shaded error bar: 95% confidence interval.

**
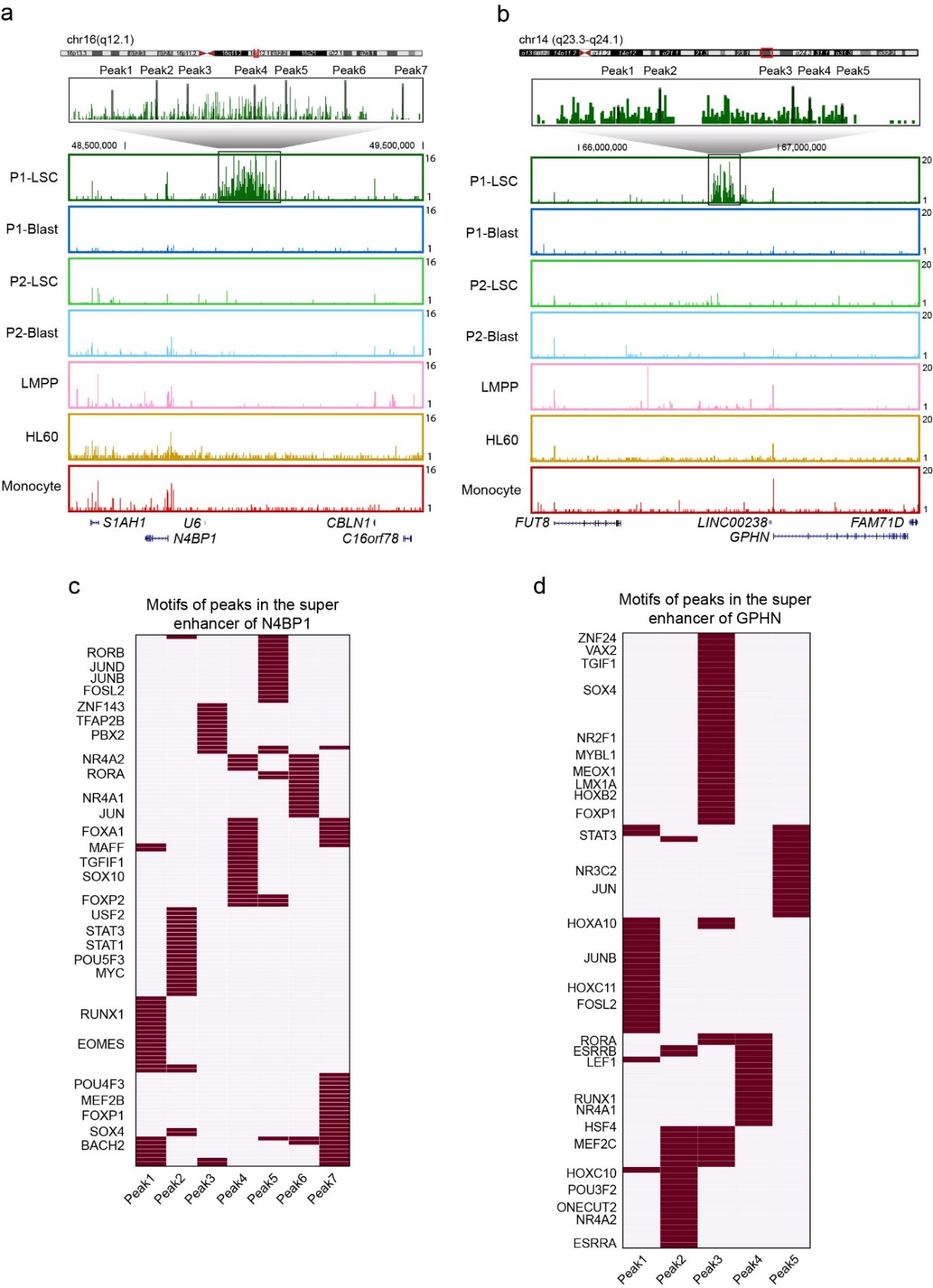
**

**Figure S3.** Super-enhancers predicted by APEC for the scATAC-seq data of cells from AML patients. (**a, b**) The genome browser track shows the aggregated scATAC-seq signal of the super-enhancer of P1-LSC cells upstream of *N4BP1* (a) and *GPHN* (b). (**c, d**) The motifs associated with peaks in the super-enhancer upstream of *N4BP1* (c) and *GPHN* (d).

**
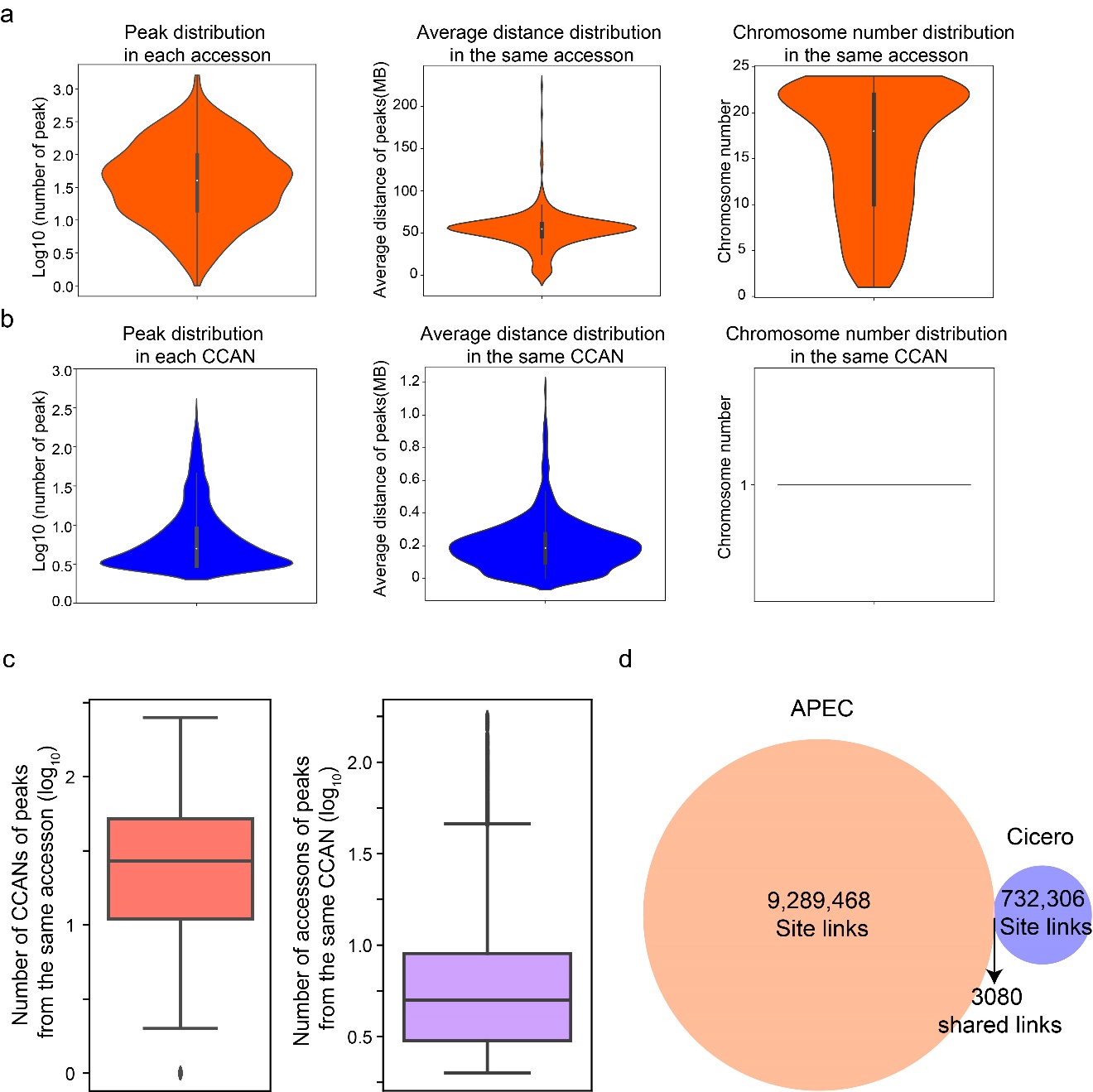
**

**Figure S4.** Comparison of the peak grouping algorithms used by APEC and Cicero on the hematopoietic dataset. (**a**) The characteristics of accessons in APEC. Left panel: distribution of peaks in each accesson; middle panel: genomic distances of peaks belong to the same accesson; right panel: number of chromosomes with peaks belong to the same accesson. (**b**) The characteristics of CCAN (defined by Cicero), as in (a). (**c**) The distribution of the number of CCANs of peaks from the same accesson (left), and the distribution of the number of accessons of peaks from the same CCAN (right). (**d**) Site links discovered by APEC and Cicero.

**
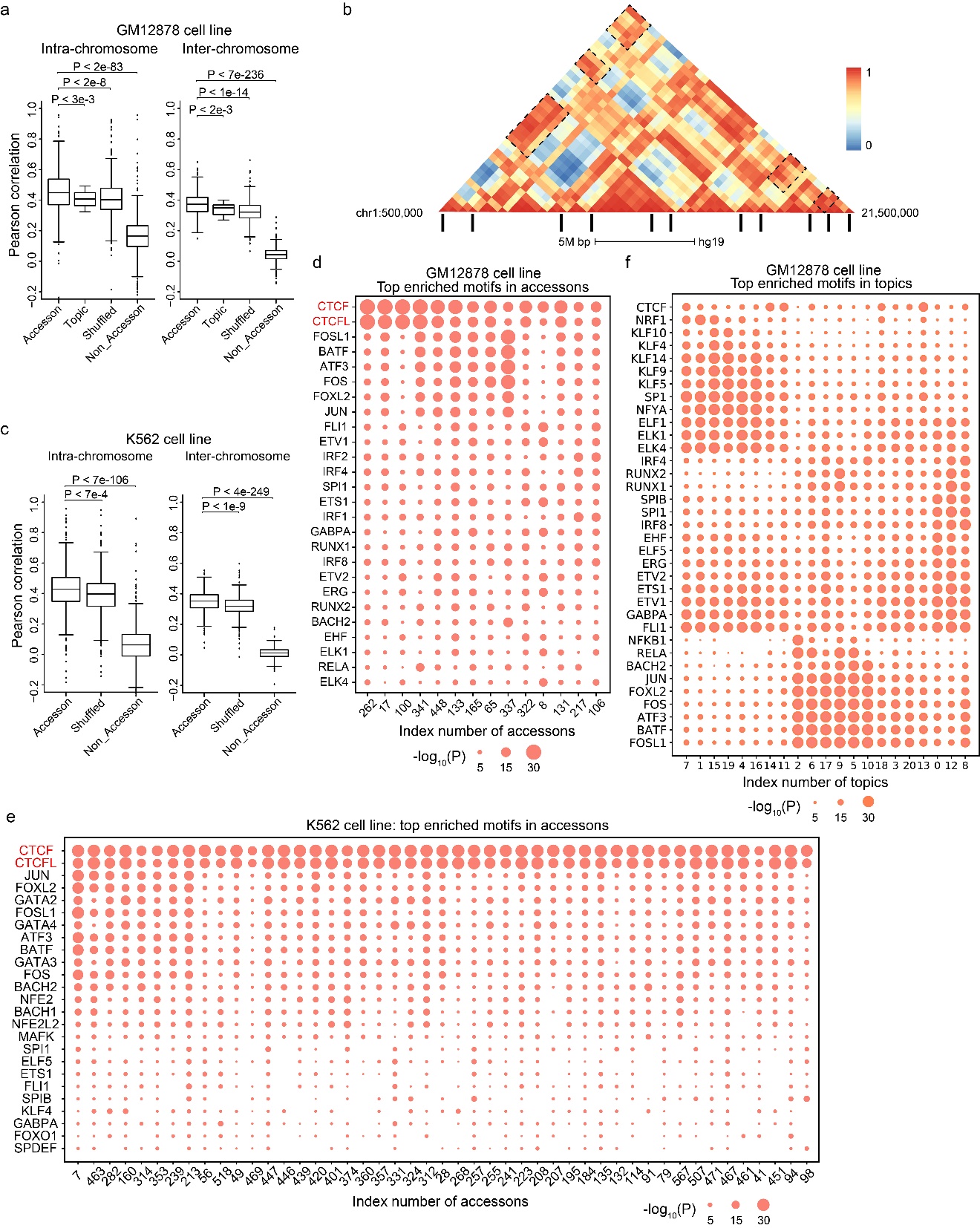
**

**Figure S5**. (**a**) Box plots presenting the average spatial distance of peaks in the same accesson or topic versus randomly shuffled peaks, and non-accessible genomic regions in the GM12878 cells. Spatial distance was estimated from chromosome conformation capture (Hi-C) technology. Left panel: Hi-C correlation of intra-chromosomal windows; right panel: Hi-C correlation of inter-chromosomal windows. (**b**) The Hi-C profile of genomic regions between chr1:500,000-21,500,000 in GM12878 cells. The black bars below the Hi-C track denote peaks in the same accesson from APEC. Dotted boxes indicate examples of peaks in the same accesson that are distant in genomic positions but close in space. (**c**) Box plots presenting the average spatial distance between peaks in the same accesson versus randomly shuffled peaks and non-accessible genomic regions in K562 cells. (**d, e**) Top enriched motifs in the accessons with more than 500 peaks, in GM12878 (d) and K562 (e) cells. (**f**) Top enriched motifs of peaks in topics in GM12878 cells.


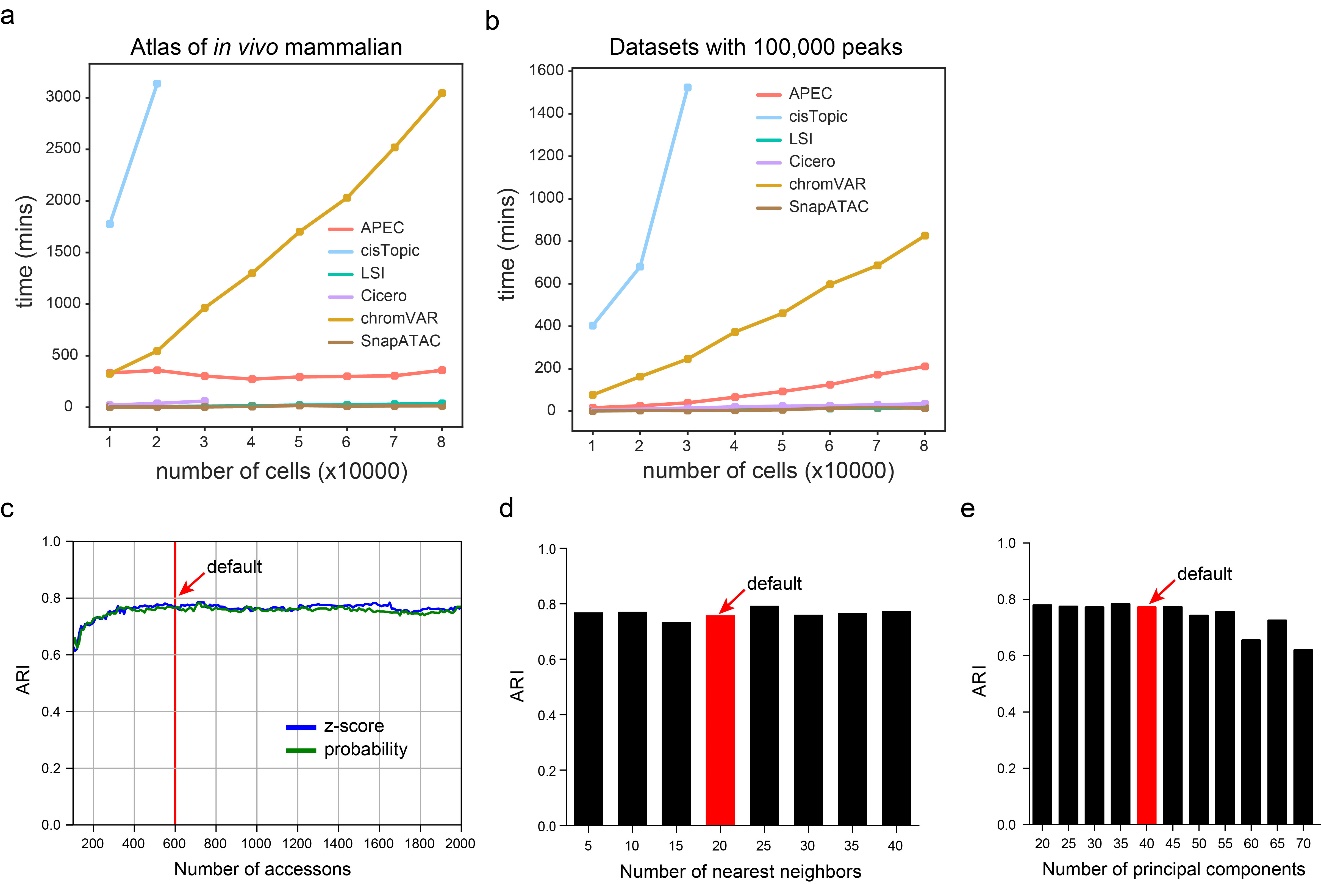


**Figure S6.** (**a, b**) The computing time required for different algorithms to cluster cell numbers from 10,000 to 80,000 with all peaks (a) and 100,000 peaks (b). The data were sampled from the single-cell atlas of *in vivo* mammalian chromatin accessibility. CisTopic was performed using 8 CPU threads and all the other tools with 1 CPU thread. (**c-e**) The ARI values of the clustering results that used different numbers of accessons (c), nearest neighbors (d), and principle components (e). The dataset includes the cells from two AML patients and three cell lines. Default values are noted in red.


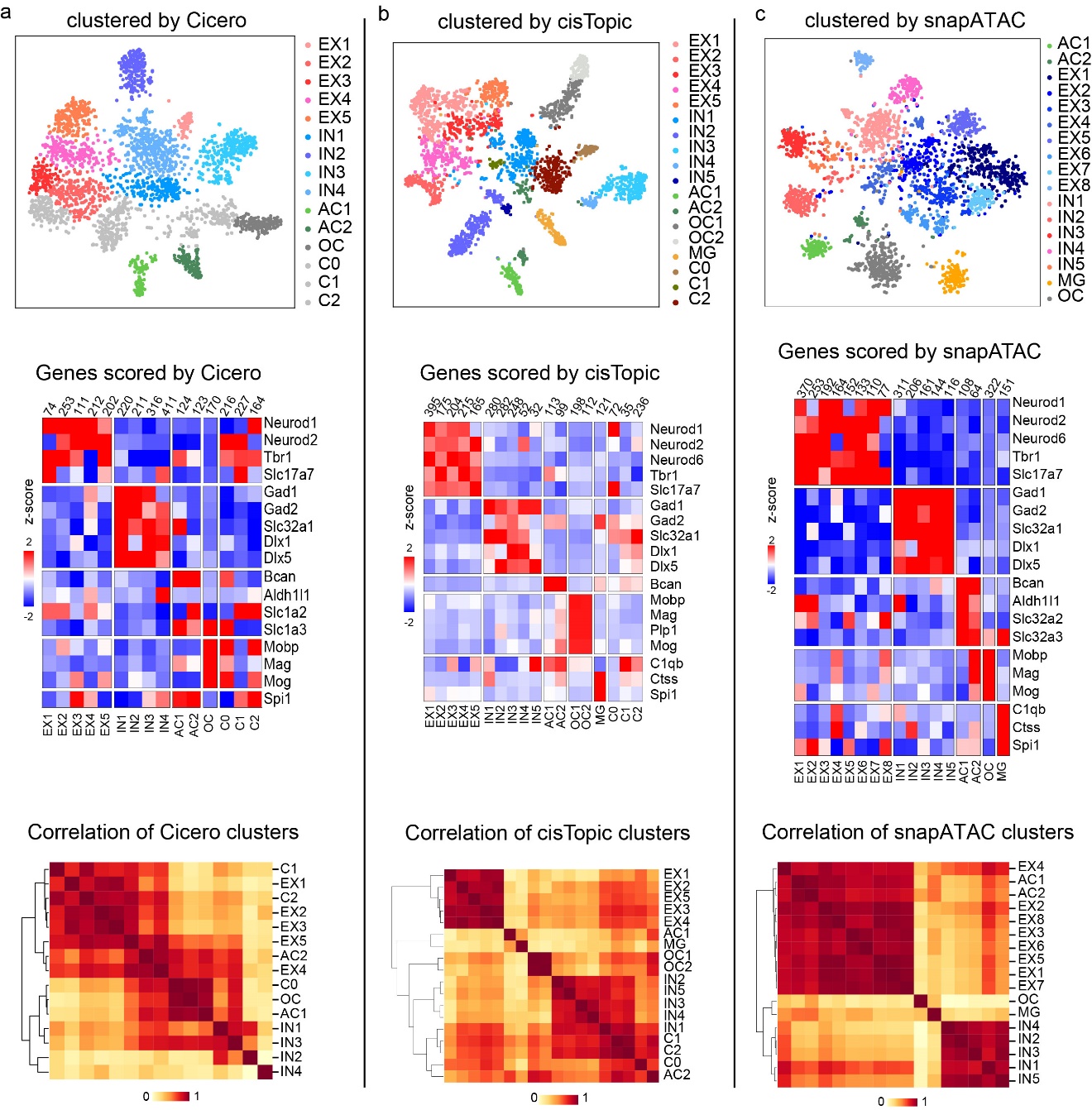


**Figure S7.** (**a**) The clustering and cell-type classification of the mouse forebrain dataset by Cicero. Upper panel: cell clusters obtained by Cicero, illustrated in the tSNE diagram. Middle panel: the z-scores of the average gene scores of cell clusters, obtained by Cicero. Lower panel: the hierarchical clustering of the Pearson correlations between cell clusters identified by Cicero. (**b, c**) The clustering and cell-type classification of the same dataset by cisTopic and SnapATAC respectively, as in (a).

**
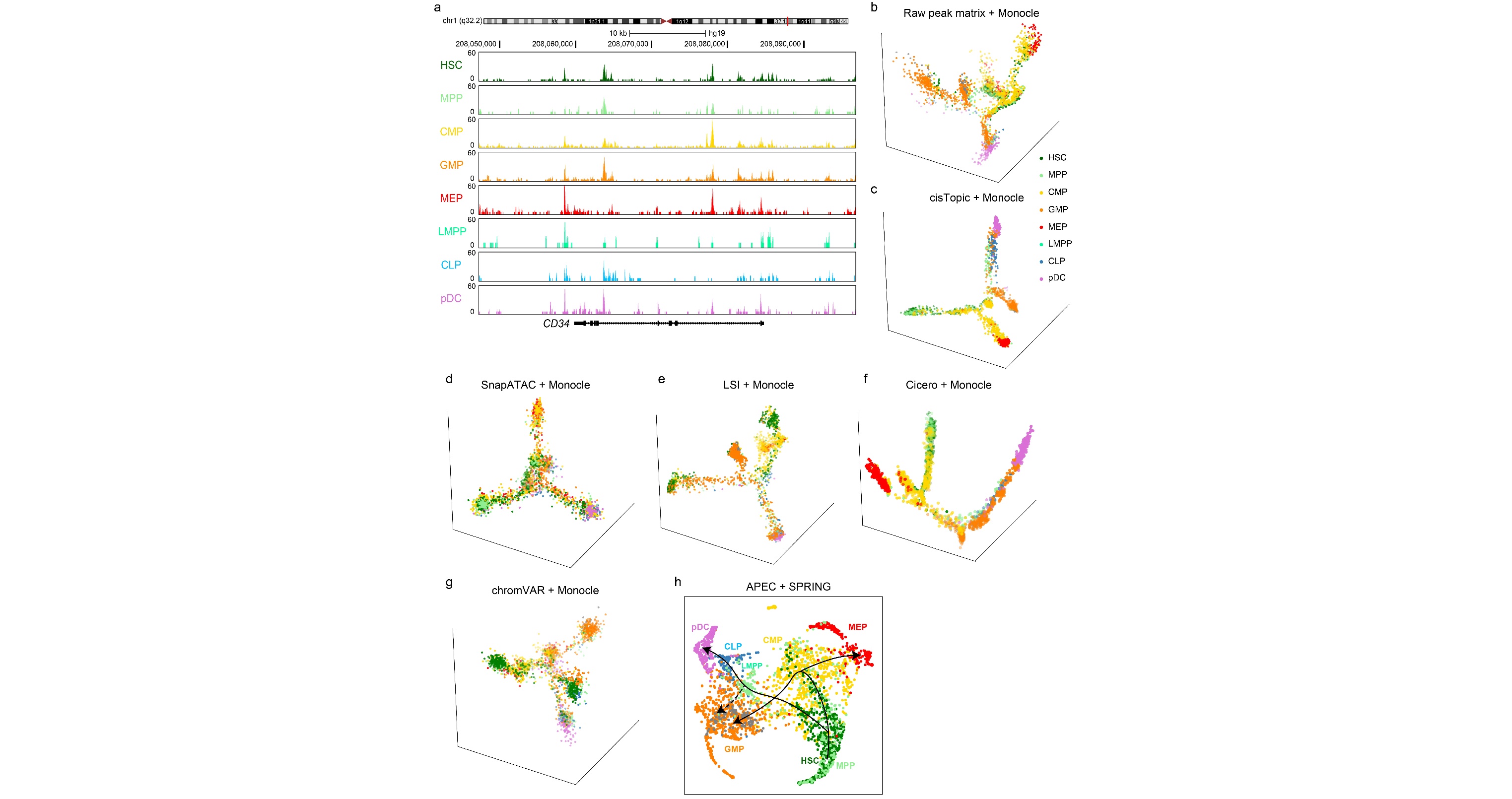
**

**Figure S8.** (**a**) UCSC genome browser track diagram of the normalized fragment count around gene *CD34* for each hematopoietic cell type. (**b-g**) The pseudotime trajectories constructed by the combination of Monocle and the raw peak count matrix, the topic matrix from cisTopic, the normalized count matrix from SnapATAC, the LSI matrix, the aggregated model matrix from Cicero, and the bias corrected deviation matrix from chromVAR, respectively. (**h**) The pseudotime trajectory constructed by the combination of SPRING and the accesson matrix from APEC.

**
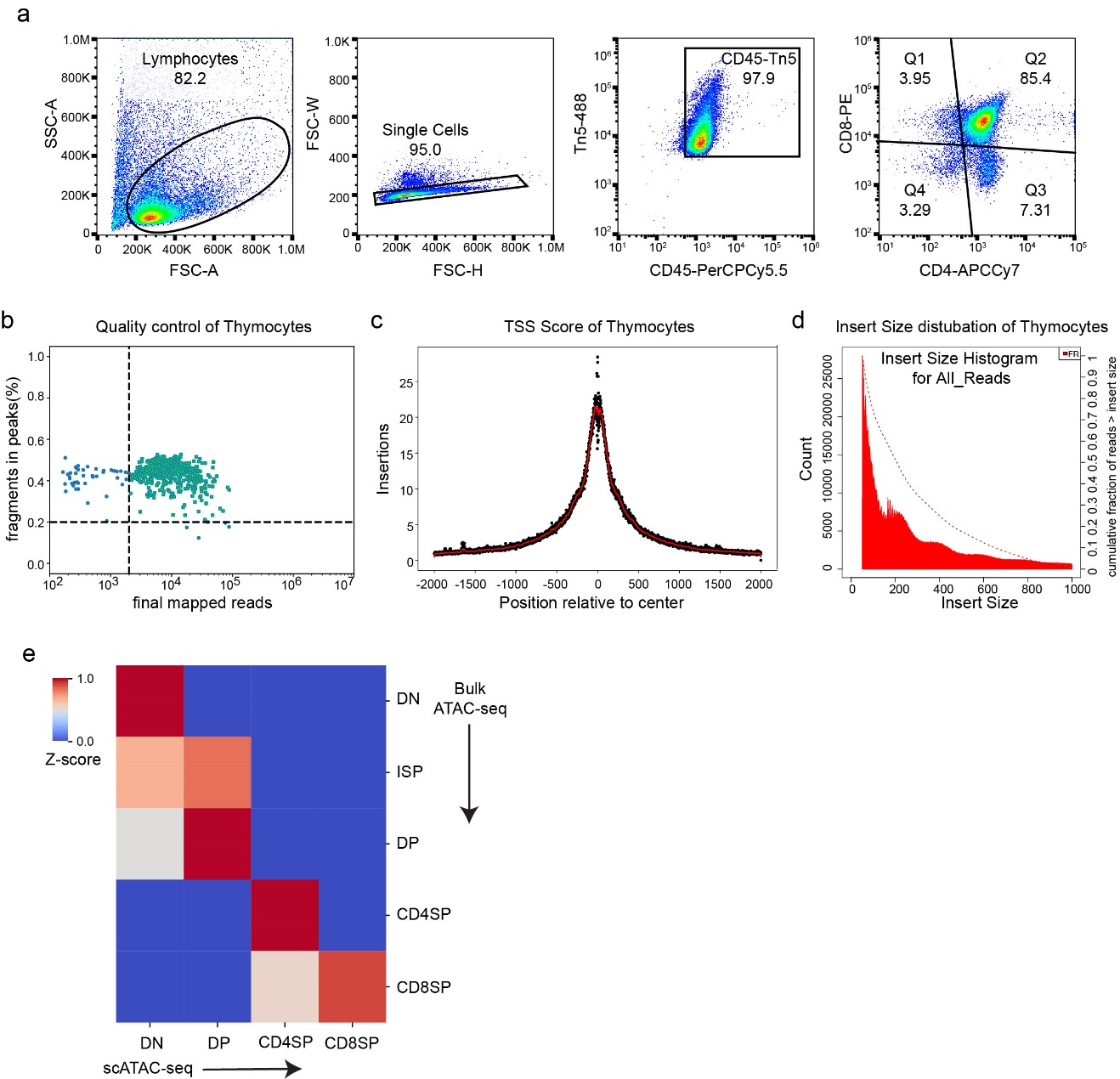
**

**Figure S9.** (**a**) Gating strategy of the mouse thymocytes in ftATAC-seq. (**b-d**) Quality control diagrams for the mouse thymocyte data, including reads numbers and percentage of fragments in peaks for each cell (b), average count of scATAC-seq insertions around TSS regions (c), and statistical distribution of fragment lengths (d). (**e**) The z-score of correlation between the cell types from ftATAC-seq and bulk ATAC-seq data.

**
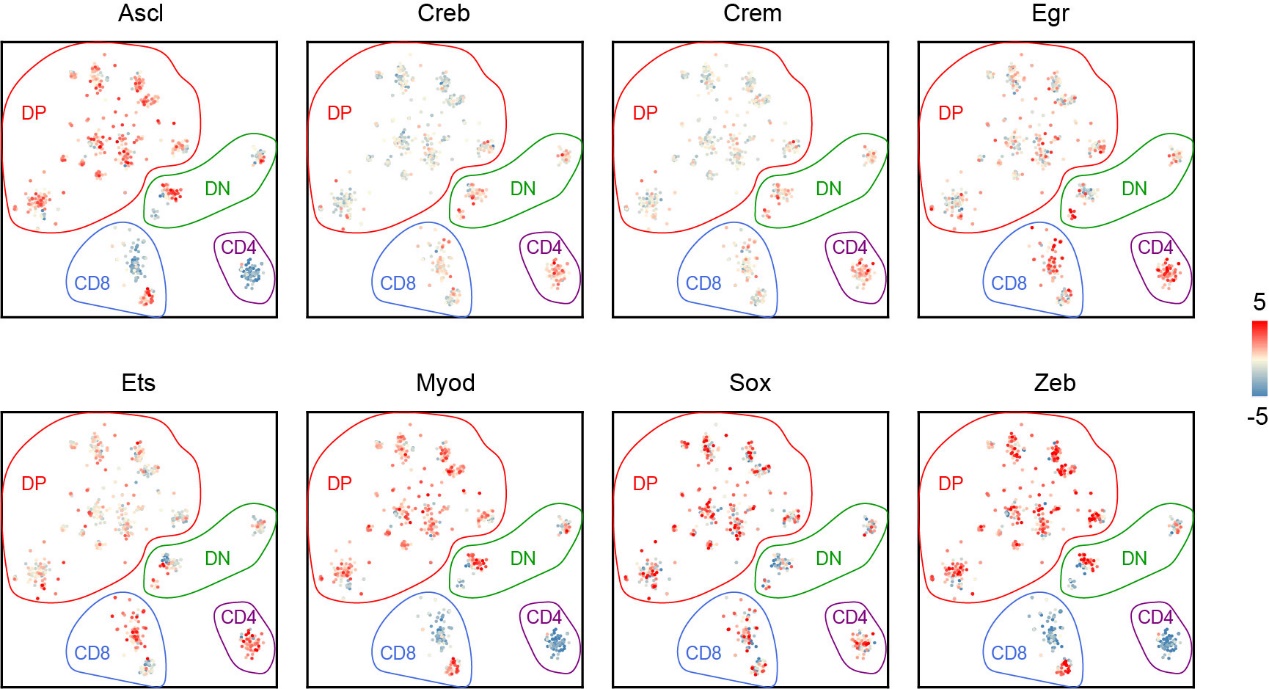
**

**Figure S10.** Selected significant motifs enriched in different thymocyte subtypes obtained by the APEC algorithm.

**
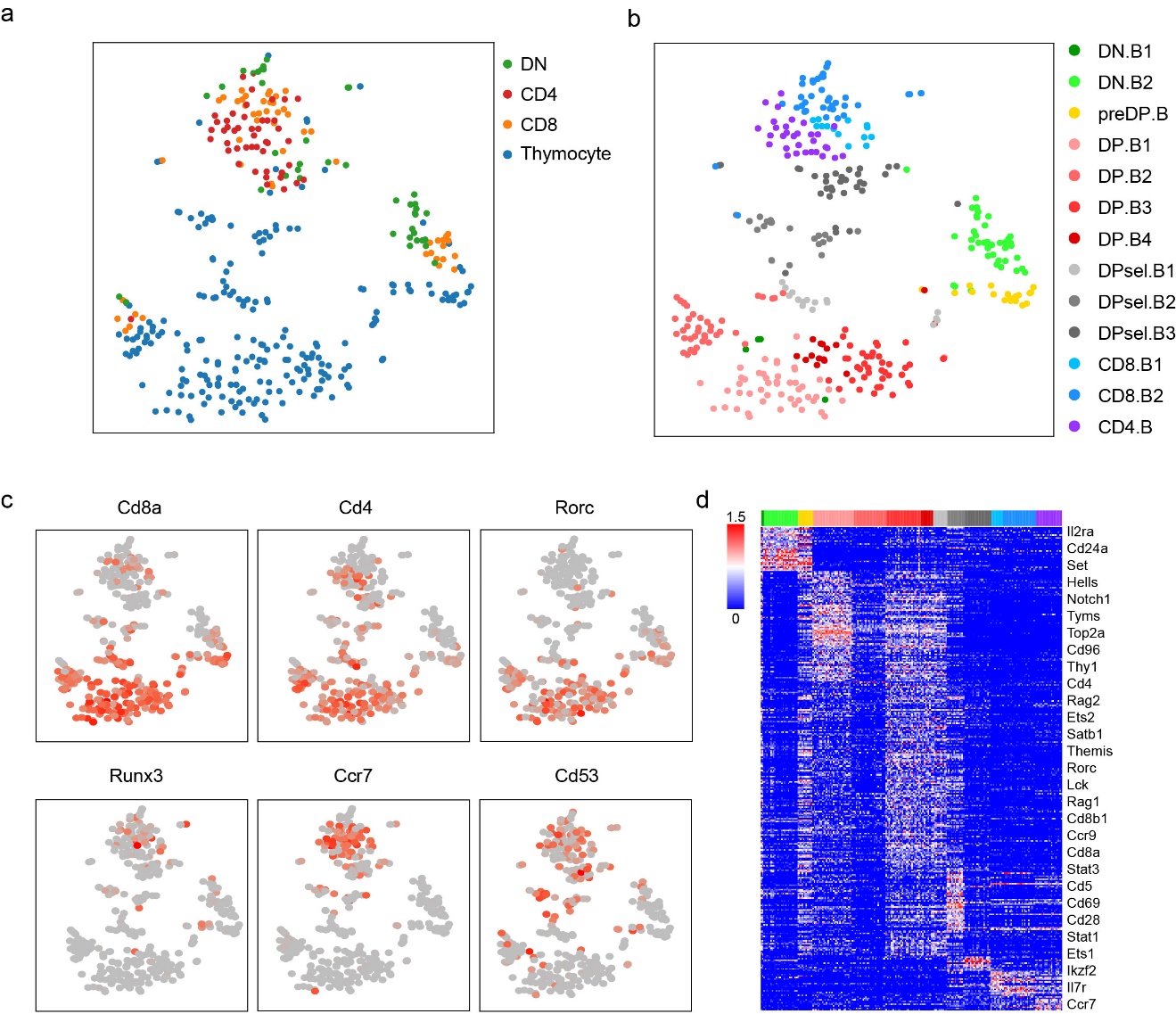
**

**Figure S11.** Single-cell transcriptome analysis of *Mus musculus* thymocytes from SMART-seq. (**a**) tSNE diagram of the single-cell expression matrix of *Mus musculus* thymocytes, labeled by the FACS index of each cell. (**b**) Louvain clustering of the same single-cell dataset obtained by Seurat. The cell types of these clusters were classified by the expression of corresponding marker genes. (**c**) Important marker genes were differentially expressed in different cell clusters. (**d**) Heatmap of the expressions of all genes significantly differentially expressed between cell clusters. The top color bar used the same scheme described in (b) to render cells of different clusters.

**
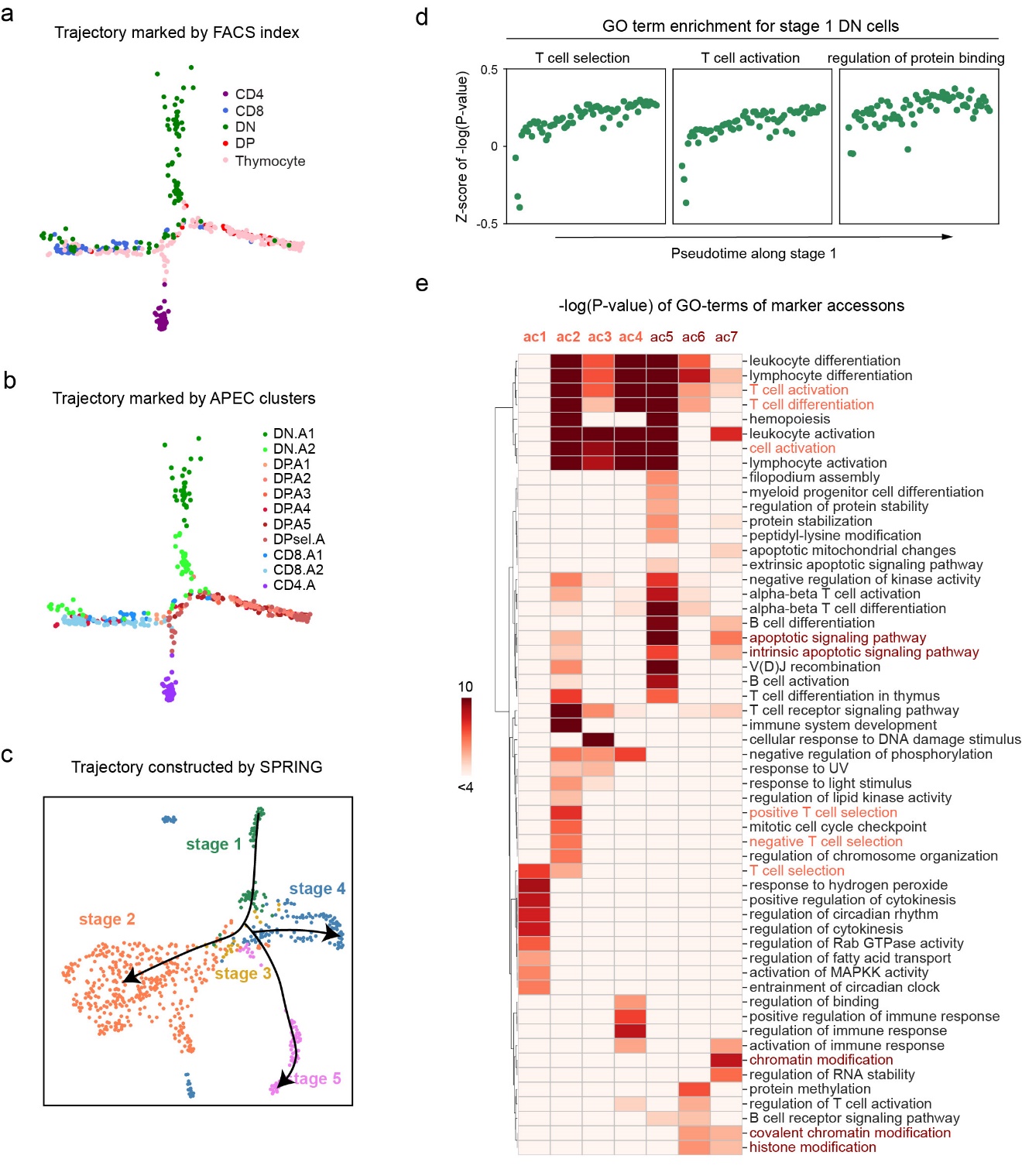
**

**Figure S12.** Developmental characteristics of single-cell samples captured by APEC. (**a, b**) Pseudotime trajectory of scATAC-seq data from *Mus musculus* thymocytes labeled with the FACS index and APEC cluster index. (**c**) Pseudotime trajectory constructed by applying SPRING to the accesson matrix. The colors of cells denote their stages in the APEC trajectory results. (**d**) Z-scores of the -log(P-value) of the GO terms along the pseudotime trajectory of stage 1 cells. (**e**) Logarithm of the P-value of GO terms searched from peaks in accessons ac1~ac7, which are the marker accessons of cluster DP. A1 and DP. A3/4/5 of thymocytes.
