# Supplementary material for: APEC: an accesson-based method for single-cell chromatin accessibility analysis
Source: Genome Biol. 2020 May 12;21:116. doi: 10.1186/s13059-020-02034-y (PMC7218568; doi:10.1186/s13059-020-02034-y)
Supplement: Supplementary file 5 — Additional file 5. Review history. [file 13059_2020_2034_MOESM5_ESM.docx]

Review History

**First round of review**

**Reviewer 1**

**Were you able to assess all statistics in the manuscript, including the appropriateness of statistical tests used?**

Yes

**Were you able to directly test the methods?**

Yes

**Comments to author:**

The manuscript describes APEC, a bioinformatics tool that analyzes scATAC-seq data towards characterizing cell development.  The key premise is that accessons are features that can better represent cell regulatory states than features extracted by other similar programs such as cicerio, cistopics, etc. and have the capability of capture long-range interactions.  It is impressive that the authors leverage many datasets in a variety of application scenarios, not just cell type profiling but also pseudotime inference, trajectory construction, integration with scRNA-seq data, etc. in several interesting biological processes such as hematopoiesis, forebrain/thymocyte development, etc. They also described a new experimental technique ftATAC-seq that can improve data quality by fluorescently gating out low quality cells and demonstrated its efficiency in characterizing thymocyte heterogeneity.  Overall, the work is fairly comprehensive in terms of scope and potential broad impact.  The software appears reliably implemented.

However, the work can be further improved in terms of rigor and writing
1.     Comparisons with other programs were not done in systematic ways.  For example,
*     the methodology of cisTopic was never properly described, even although its performance in cell-type clustering appeared similar to that of APEC.
*     The point that accessons may have captured valid long-range relations was not emphasized enough, as it is the key feature that delineates APEC from other programs.  Orthogonal evidences such as Hi-C and motif-multiplicity need to be made more visible.  
*     The small overlap (1%) with Cicero in peak pairing is not well justified, given that the methods have similar assumptions (just different ways of grouping peaks).
*     ATAC/RNA integration (Fig. 2 and Fig. 4h) should compare with other available integration methods such as Seurat.
2.     The writing is largely acceptable but there are quite some grammatical and spelling errors.  The descriptions tend to be technical, not focused on scientifically interesting information/logics. For example,
*     In Introduction, why performing this work was only supported by one statement: "great challenges still remain for current algorithms to precisely cluster large number of cells and predict gene expressions from single cell chromatin accessibility profiles".  That does not provide a strong scientific motivation for performing such an extensive investigation.
*     Labels from reference studies are often conveniently regarded as the "gold standard" with no additional scientific justifications.  The labels in the original publications could be wrong too. It would be helpful if the authors can cite cell development literature (e.g., physiology, morphology, ontology) that better supports such validations.
*     Lack accurate descriptions on the variety of scATAC-seq technologies used in different studies.  Did they all use the same technology developed by 10X Genomics? Are there differences in chemistry or protocols?  Without these, users may be confused when they can use APEC.

**Reviewer 2**

**Were you able to assess all statistics in the manuscript, including the appropriateness of statistical tests used?**

Yes

**Were you able to directly test the methods?**

No

**Comments to author:**

In this paper Bin Li et al introduced APEC to classify each cell in Single Cell ATAC-seq experiment. They looked into the change of signals across all the peaks and combined individual peaks into groups called accessions where the peaks have same signal fluctuation. These accessions are generated without relying on any prior information. Authors have applied APEC to multiple datasets of single cell ATAC-seq and have demonstrated its efficiency in clustering cell-types. APEC is also capable of identifying super enhancers, search for motifs, score gene activity, integrate single cell RNA-seq with ATAC-seq and infer psuedotime trajectory by integrating it with monocle. Performance of APEC in determining developmental trajectory was shown by successfully inferring the regulome dynamics of the thyme development process. Overall this is an important work given how few methods/tools are available that supports scATAC-seq analysis. However, I feel that authors need to address some outstanding issues before ready for publication

Comments to authors:

1. To show the scalability of APEC, the authors used the dataset of single cell mouse chromatin accessibility atlas of Cusanovich et. al. Cell (2018) DOI:https://doi.org/10.1016/j.cell.2018.06.052 with 81,173 single cells to cluster cells into their corresponding cell types . However, I am not clear how such large dataset was analyzed. Chen H. et. al. mentioned in their paper, Assessment of computational methods for the analysis of single-cell ATAC-seq data DOI: https://doi.org/10.1186/s13059-019-1854-5 , that majority of the methods they compared failed to load this large dataset even with 1 TB of RAM memory. Those were the same methods used in this paper as well. The only tool that could process the data in reasonable time was snapATAC as it used a file format build based on HDF5 that supports out of core computation by progressively loading only the data required at a certain time point. How did the authors processed and analysed this large dataset using these tools. Also, Figure S5 (C) does not show the performance of snapATAC in the comparison, but this tool is included in other comparisons. I would like to see the result of this tool in the comparison as it has been reported to be the best performing tool for this large dataset.

2. What implementation approach ensured scalability of APEC? Did they use approaches like HDF5 or some other ones? This needs to be discussed in the method section.

3. The authors demonstrated performance of APEC in low sequencing coverages, but they did not mention its performance at difference noise levels.

4. APEC pipeline calls peaks on a merged single cell profile. But such peak calling on pseudobulk data lack the ability to reveal regulatory elements in rare cell populations that are significantly underrepresented in the aggregated data. This is a critical concern as Rongxin Fang et. al. explained in their paper in terms of brain cells, where a cell population representing less than 1% of cell population still plays critical role in neural circuit. How the authors would address this issue in APEC?

5. In the computational method assessment paper, Chen H. et. al. explained that methods which preserve information at the peak/bin level performs better in distinguishing cell-types compared to TF-motif level or gene-centric level summarisation. Their interpretation for this was that the individual peaks are able to capture the complexity of one regulatory circuits more precisely. But APEC still outperforms the peak/bin based method (for eg. snapATAC) even though it is grouping peaks based on their synergistic signal patterns. What could be the possible reason behind this?

6. When there would be minor clusters along with major clusters, for eg. with Cusanovich dataset, how APEC would perform? Although the authors have shown this in Figure 1(d) and S1, it was not obvious how the clustering was performed.

7. How APEC would correct for batch effects if there is any and what would be it's performance then?

**Authors Response**

**Point-by-point responses to the reviewers’ comments:**

*We thank the reviewers for their positive assessments and thoughtful suggestions in improving this work. Point-by-point responses are as follows.*

*We have revised our manuscript mainly in the following aspects:*

*Ensure the manuscript conforms to meet the style of Method article style, including shortened the Abstracts to meet the words limitation and merged the Methods section to manuscript.*

*Compared APEC with other state-of-the-art methods used to analyze scATAC-seq datasets, including cisTopic and SnapATAC as the reviewers suggested.*

*We added several paragraphs in Introduction, Results, Discussion and Methods sections to better explain the motivations, advantages and limitations and methodologies of our study. Meanwhile revised figures and figure legends to address the reviewers’ questions, including Figure S2, S4, S5, S6, S7 & S8.*

Reviewer #1 (Remarks to the Author):

The manuscript describes APEC, a bioinformatics tool that analyzes scATAC-seq data towards characterizing cell development. The key premise is that accessons are features that can better represent cell regulatory states than features extracted by other similar programs such as cicerio, cistopics, etc. and have the capability of capture long-range interactions. It is impressive that the authors leverage many datasets in a variety of application scenarios, not just cell type profiling but also pseudotime inference, trajectory construction, integration with scRNA-seq data, etc. in several interesting biological processes such as hematopoiesis, forebrain/thymocyte development, etc. They also described a new experimental technique ftATAC-seq that can improve data quality by fluorescently gating out low quality cells and demonstrated its efficiency in characterizing thymocyte heterogeneity. Overall, the work is fairly comprehensive in terms of scope and potential broad impact. The software appears reliably implemented.

*Response: We thank the reviewer for their positive assessment that our work is impressive and overall fairly comprehensive in terms of scope and potential broad impact, as well as their constructive suggestions to improve our manuscript.*

However, the work can be further improved in terms of rigor and writing:

1.	Comparisons with other programs were not done in systematic ways. For example,

the methodology of cisTopic was never properly described, even although its performance in cell-type clustering appeared similar to that of APEC.

*Response: In our previous submission, we have compared APEC with all the other state-of-the-art single cell chromatin accessibility analysis methods, including Latent Semantic Indexing (LSI) [1, 2], cisTopic [3], Cicero [4], chromVAR [5] and SnapATAC [6], and performed extensive benchmarking over all the methods as much as we could in terms of the accuracy of single-cell clustering, robustness and stability, speed and scalability under limited computational resources (1 CPU thread and 512 GB RAM), and pseudotime trajectory prediction (Fig. 1, 2, 3 and Figure S2, S6, S7, S8).*

*Among these methods, cisTopic is one of the best tools for cell-type clustering, although it is usually more than 10 times slower than the other tools (revised Figure S6a&b). CisTopic was a published approach and that was why we did not describe its methodology in detail in our previous submission. In this revised manuscript, we added a paragraph in Results (highlighted in the main text) to describe its methodology, as follows:*

*“In cisTopic, hundred thousands of peaks were aggregated into dozens of representative ‘topics’ (usually 10~40) that preserve the chromatin accessibility information of each cell via a Bayesian topic modeling method. Cells were then clustered based on their distance in the “topic” space.”*

*In addition, we compared APEC with cisTopic on the peaks in “accessons” versus those in “topics” of the GM12878 dataset (revised Figure S5a, d, f). By integrating the chromatin conformation profiles from Hi-C experiments with the scATAC-seq profile on the same cells, we found that peaks in the same “accesson” were spatially slightly closer to each other than the representative peaks in the same “topic”. Motif enrichment analysis also suggested that peaks in “accessons” were spatially more coherent than those in “topics”, since the CTCF and CTCFL motifs were top enriched in “accessons” but not in “topics” (revised Figure S5d&f). We believe that these may be the main reasons why APEC provides slightly better cell clustering than cisTopic.*

*Figure S5. (a) Box plots presenting the average spatial distance of peaks in the same accesson or topic versus randomly shuffled peaks, and non-accessible genomic regions in the GM12878 cells. Spatial distance was estimated from chromosome conformation capture (Hi-C) technology. Left panel: Hi-C correlation of intra-chromosomal windows; right panel: Hi-C correlation of inter-chromosomal windows. (b) The Hi-C profile of genomic regions between chr1:500,000-21,500,000 in GM12878 cells. The black bars below the Hi-C track denote peaks* *in the same accesson from APEC. Dotted boxes indicate examples of peaks in the same accesson that are distant in genomic positions but close in space. (c) Box plots presenting the average spatial distance between peaks in the same accesson versus randomly shuffled peaks and non-accessible genomic regions in K562 cells. (d, e) Top enriched motifs in the accessons with more than 500 peaks, in GM12878 (d) and K562 (e) cells. (f) Top enriched motifs of peaks in topics in GM12878 cells.*

The point that accessons may have captured valid long-range relations was not emphasized enough, as it is the key feature that delineates APEC from other programs. Orthogonal evidences such as Hi-C and motif-multiplicity need to be made more visible.

*Response: We thank the reviewer for their constructive suggestions. To emphasize the point that accessons may have captured valid long-range relations between peaks, we performed two additional analyses in the revised manuscript: (1) We integrated the scATAC and Hi-C profiles from K562 cell line and performed the same analysis as we did for GM12878 in the revised Figure S5a. Within expectation, the Hi-C correlations of peaks in the same accesson were significantly higher than those randomly selected, which further confirmed our conclusion that peaks in the same accesson were spatially much closer to each other (noted as revised Figure S5c). (2) Since CTCF is a well-known architectural protein that mediates long-range chromatin looping and bridges genome topology and function [7-11], if APEC does group peaks that are spatially adjacent with each other, the CTCF motif should be enriched in peaks from the same accesson. As expected, we found that the CTCF/CTCFL motifs were the top two enriched motifs in accessons with more than 500 peaks (revised Figure S5d&e), which strongly supported our conclusion that accessons may have captured valid long-range relations. We added these discoveries in the revised Results.*

The small overlap (1%) with Cicero in peak pairing is not well justified, given that the methods have similar assumptions (just different ways of grouping peaks).

*Response: As we explained in the main text and Figure S4a&b, APEC and Cicero are two completely different approaches: (1) APEC groups synergistic peaks genome-wide, but Cicero groups peaks within a certain range of genomic distance (default: 500 kb), termed cis-co-accessibilities networks (CCAN). (2) APEC defines the similarity of peaks based on the correlation of peak fragment counts across all the single cells, while Cicero uses the average signal of each cell-cluster as the signal for each peak, and then calculates the correlation between the peaks. Therefore, even on the same dataset, APEC and Cicero end up with very different “peak groups”. On average each accesson contained -40 peaks (median number) which were distributed to -30 CCANs, and -5 peaks were found in each CCAN and distributed to -5 accessons (revised Figure S4c), which results in a small overlap in peak paring.*

*Figure S4. Comparison of the peak grouping algorithms used by APEC and Cicero on the hematopoietic dataset. (a) The characteristics of accessons in APEC. Left panel: distribution of peaks in each accesson; middle panel: genomic distances of peaks belong to the same accesson; right panel: number of chromosomes with peaks belong to the same accesson. (b) The characteristics of CCAN (defined by Cicero), as in (a). (c) The distribution of the number of CCANs of peaks from the same accesson (left), and the distribution of the number of accessons of peaks from the same CCAN (right). (d) Site links discovered by APEC and Cicero.*

ATAC/RNA integration (Fig. 2 and Fig. 4h) should compare with other available integration methods such as Seurat.

*Response: Since the cells in the mouse thymocytes dataset were labeled with their cell types (DN, DP, CD8+, CD4+) by FACS experiment, we used this dataset as a benchmark to compare the performance of APEC and Seurat to intergrade ATAC/RNA profiles. The correlation analysis of cell types predicted from scATAC and scRNA data by Seurat indicated that DP (DP.A1~5) and CD4+ (CD4.A) T cells in scATAC data were mostly identified as DP and CD4+ T cells from scRNA data respectively, suggesting that Seurat successfully integrated scATAC and scRNA on these cell types (Graph 1a). However, the DN (DN.A1), DPsel (DPsel.A) and CD8+ (CD8.A1) T cells were mixed-classified, compare to the integration from APEC (Figure 4h). Same analysis was also performed on neuronal cells (Graph 1b&c) and our results suggested that Seurat might not to be suitable for integration of these data set, while APEC gave more reasonable integrating results.*

*Here we would like to clarify that the current version of APEC was not capable to anchor each cell from scATAC to those from scRNA at a single cell resolution, rather the ATAC/RNA integration was evaluated on a group of cells (i.e. cell subtypes). We acknowledge that integrating multi-omics data at a single cell level still is a great challenge, and lots of improvements of APEC are needed to fully address this question. Since ATAC/RNA integration was not the main goal of this study, we will not include this result in the main text, but discussed in the revised manuscript instead.*

*Graph 1. Integration of scRNA and scATAC data using Seurat. (a) Percentage of cells in each scATAC cluster corresponding to the scRNA clusters on the mouse thymocytes datasets predicted by Seurat. (b, c) Percentage of cells in each scATAC (snATAC) cluster corresponding to the scRNA (inDrops) clusters, predicted by Seurat on the mouse excitatory neurons (b) and inhibitory neurons (c) respectively.*

2.	The writing is largely acceptable but there are quite some grammatical and spelling

errors. The descriptions tend to be technical, not focused on scientifically interesting information/logics.

For example,

In Introduction, why performing this work was only supported by one statement: "great challenges still remain for current algorithms to precisely cluster large number of cells and predict gene expressions from single cell chromatin accessibility profiles". That does not provide a strong scientific motivation for performing such an extensive investigation.

*Response: We thank for the reviewer’s suggestion and added the following paragraph in the revised manuscript to better rationalize the motivation of our study:*

*“However, great challenges still remain for current algorithms to provide comprehensive analysis on single cell chromatin accessibility profiles. For instance: (1) how to improve the accuracy of single cell clustering on cells with minor difference; (2) how to better illuminate the cellular developmental processes based on scATAC-seq data; (3) how to integrate RNA/ATAC profiles and predict the RNA expressions from scATAC-seq data and further reveal the biological insights of the system; and (4) how to manipulate massive sequencing data with high speed, scalability and stability. Therefore, a fast, scalable and stable algorithm is needed to precisely categorize cell subgroups, to predict gene expressions and developmental trajectories, and thereby to provide a deeper mechanistic understanding of single-cell epigenetic heterogeneity and regulation.”*

Labels from reference studies are often conveniently regarded as the "gold standard" with no additional scientific justifications. The labels in the original publications could be wrong too. It would be helpful if the authors can cite cell development literature (e.g., physiology, morphology, ontology) that better supports such validations.

*Response: Thanks for your kind advise. We checked the sorting strategy in the original reference study on HSC development. According to the literature, the FACS labelling of the HSC developmental subtypes were confirmed at both the bulk and single-cell levels [23, 24]. Besides, the labelling for the CD4 and CD8 cells used in our ftATAC-seq experiment were well accepted as the standard markers during thymocyte development [25], and were also confirmed at single-cell level by recent study [26]. We thereby cited these references in the revised manuscript to better support the FACS labeling.*

Lack accurate descriptions on the variety of scATAC-seq technologies used in different studies. Did they all use the same technology developed by 10X Genomics? Are there differences in chemistry or protocols? Without these, users may be confused when they can use APEC.

*Response: Researchers have developed a variety of protocols to acquire single cell chromatin accessibility with benefits at different aspects, such as scATAC [27], snATAC [14, 28] , sciATAC [1, 29] , Pi-ATAC [30] and in this study the ftATAC and etc. These scATAC-seq technologies may use different chemistry or protocols, but will end up with similar data profiles. As a software, APEC was not developed for any specific type of data, rather it is generally applicable to a variety of scATAC-seq profiles. We have tested datasets from all of the aforementioned scATAC-seq techniques and APEC worked well for all of them. For example, data used in Figure 1 was generated from Fludigm C1 chip; data used in Figure 2 was generated with the snATAC protocol; data used in Figure 4-5 was generated with our ftATAC protocol.*

*In terms of 10X Genomics, by the time this study was finished, commercial 10X Genomics kits for scATAC-seq were just available. In this revision, we tested several datasets downloaded from the 10X Genomics official website, and found that APEC worked well with the data generated from the 10X platform. We upgraded the APEC software by adding a preprocessing subroutine named “convert.convert_10X” for researchers’ convenience to use APEC to analyze scATAC-seq data from the 10X platform.*

*We added a few sentences in the revised Discussion to clarify that APEC is generally applicable.*

Reviewer #2 (Remarks to the Author):

In this paper Bin Li et al introduced APEC to classify each cell in Single Cell ATAC-seq experiment. They looked into the change of signals across all the peaks and combined individual peaks into groups called accessions where the peaks have same signal fluctuation. These accessions are generated without relying on any prior information. Authors have applied APEC to multiple datasets of single cell ATAC-seq and have demonstrated its efficiency in clustering cell-types. APEC is also capable of identifying super enhancers, search for motifs, score gene activity, integrate single cell RNA-seq with ATAC-seq and infer psuedotime trajectory by integrating it with monocle. Performance of APEC in determining developmental trajectory was shown by successfully inferring the regulome dynamics of the thyme development process. Overall this is an important work given how few methods/tools are available that supports scATAC-seq analysis. However, I feel that authors need to address some outstanding issues before ready for publication.

*Response: We thank the reviewer for their positive comments that APEC is an important tool for scATAC-seq analysis, as well as constructive suggestions for improving the current manuscript.*

1. To show the scalability of APEC, the authors used the dataset of single cell mouse chromatin accessibility atlas of Cusanovich et. al. Cell (2018) DOI: <https://doi.org/10.1016/j.cell.2018.06.052> with 81,173 single cells to cluster cells into their corresponding cell types. However, I am not clear how such large dataset was analyzed. Chen H. et. al. mentioned in their paper, Assessment of computational methods for the analysis of single-cell ATAC-seq data DOI: <https://doi.org/10.1186/s13059-019-1854-5,> that majority of the methods they compared failed to load this large dataset even with 1 TB of RAM memory. Those were the same methods used in this paper as well. The only tool that could process the data in reasonable time was snapATAC as it used a file format build based on HDF5 that supports out of core computation by progressively loading only the data required at a certain time point. How did the authors processed and analysed this large dataset using these tools.

*Response: APEC uses the Matrix Market (MTX) file format, which maintains only the non­zero elements in a matrix, to store and process the sparse cell-peak count matrix. APEC also adopts the “Scipy.sparse” python library to operate the data matrix with MTX format. In this way, APEC was capable to process a sparse matrix with 81,173 (cells) X 436,206 (peaks) using less than 50 GB RAM memory. Same approach was applied to test the performance of LSI by modifying the source code of the algorithm. SnapATAC and chromVAR were both able to load large dataset, but chromVAR took way much longer than SnapATAC to finish the cell clustering process (revised Figure S6a&b).*

*However, CisTopic and Cicero indeed failed to process large datasets, even on a computer with 512 GB memory, and therefore in the revised Figure. S6a we showed the performance of these two methods on dataset with up to 30,000 cells. All the tested methods except cisTopic, were capable to load the 80,000 (cells) X 100,000 (peaks) matrix on a computer with 512GB RAM (revised Figure S6b). By the way, the MTX format is also supported by the official 10X Genomics genome mapping pipeline named Cellranger. The outputs from Cellranger can be easily implemented into APEC by calling the subroutine “convert.convert_10X”, making APEC capable of analyzing scATAC-seq data from the 10X platform.*

Also, Figure S5 (C) does not show the performance of snapATAC in the comparison, but this tool is included in other comparisons. I would like to see the result of this tool in the comparison as it has been reported to be the best performing tool for this large dataset.

*Response: We compared the performance of SnapATAC with all the other tools to process large dataset and updated the results into the revised Figure S6a&b. In terms of speed and scalability, SnapATAC is one of the best among the tested tools, similar to LSI. We mentioned the performance of SnapATAC in the revised manuscript.*

*Figure S6. (a, b) The computing time required for different algorithms to cluster cell numbers from 10,000 to 80,000 with all peaks (a) and 100,000 peaks (b). The data were sampled from the single-cell atlas of in vivo mammalian chromatin accessibility. CisTopic was performed using 8 CPU threads and all the other tools with 1 CPU thread. (c-e) The ARI values of the clustering results that used different numbers of accessons (c), nearest neighbors (d), and principle components (e). The dataset includes the cells from two AML patients and three cell lines. Default values are noted in red.*

2. What implementation approach ensured scalability of APEC? Did they use approaches like HDF5 or some other ones? This needs to be discussed in the method section.

*Response: As we responded in Question 1, similar to the HDF5 format, APEC uses the MTX file format, which maintains only the non-zero elements in a matrix, to store and process the sparse cell-peak count matrix. APEC also adopts the “Scipy.sparse” python library to operate the data matrix with MTX format. In this way, APEC was capable to process a sparse matrix with 81,173 (cells) X 436,206 (peaks) using less than 50 GB RAM memory. We clarified this point in the revised Methods sections as follows:*

*“APEC uses the MTX file format, which maintains only the non-zero elements in a matrix, to store and process the sparse cell-peak count matrix, and then uses the “Scipy.sparse” library in Python to operate the MTX format matrix for peak grouping and cell clustering. With this approach APEC is able to process single cell chromatin accessibility profiles with as many as 80,000 cells and 400,000 peaks within 50GB RAM. APEC can also access the MTX output files from Cellranger, implying its well compatibility with 10X Genomics datasets.”*

3. The authors demonstrated performance of APEC in low sequencing coverages, but they did not mention its performance at difference noise levels.

*Response: Noise, also termed as "dropout", in single-cell sequencing profile severely obscure important cell-cell relationships [31, 32]. For the dataset used in Figure S2a&d, we randomly selected different percentages of the elements from the original fragment count matrix and set them to zero, to simulate the noises caused by the dropout events in single-cell experiments [31, 32]. We compared the performance of all the tools to cluster cell types at noise levels from 0 to 90%, and found that APEC still exhibited an ARI>0.7 when noise level reached 40%, indicating its reliability to cluster cell types. The software was upgraded to APEC V1.2.2. SnapATAC was not included in this comparison because the algorithm starts from mapped BAM files instead of fragment count matrix and therefore unable to estimate noises.*

*Figure S2e. (e) The average ARI values of the noised data sampled from the fragment count matrix of the dataset used in (d). The X-axis represents the percentage of noised elements in the matrix. Shaded error bar: 95% confidence interval of 20 times down-sampling.*

4. APEC pipeline calls peaks on a merged single cell profile. But such peak calling on pseudobulk data lack the ability to reveal regulatory elements in rare cell populations that are significantly underrepresented in the aggregated data. This is a critical concern as Rongxin Fang et. al. explained in their paper in terms of brain cells, where a cell population representing less than 1% of cell population still plays critical role in neural circuit. How the authors would address this issue in APEC?

*Response: The classification of rare but functional cell populations is still a great challenge in single cell data processing. We agree with the reviewer that SnapATAC developed by Rongxin Fan et. al, excels at identifying very rare cell populations by constructing the fragment count matrix from genome-wide bins. When using APEC, if the user wants to identify rare cell subtypes and meanwhile retain an accurate cell clustering and psuedotime trajectory, we recommend the following options: 1. reduce the filtering parameter (“-l” parameter in APEC_prepare_steps.sh) to keep as many peaks as possible to retain the regulatory elements from rare cell populations; and 2. substitute the cell-peak fragment count matrix to the cell-bin count matrix from SnapATAC, and then perform the downstream analyses in APEC.*

*With these modified procedures, we tested APEC on the 5,000 mouse brain cells dataset offered by Rongxin Fang et al. and downloaded the cell-bin matrix from* [*https://github.com/r3fang/SnapATAC*](https://github.com/r3fang/SnapATAC)*. We found APEC identified two Sst╋ cell subtypes, i.e.*

*the Sst╋Pvalb╋ subtype (cluster 22 in Graph 2) and the Sst╋Pvalb━ subtype (cluster 18), by setting nc=26 in clustering.cluster_byAccesson of APEC V1.2.2. The two cell subtypes consist 1.3% and 0.9% of all the cells, respectively, suggesting that APEC was capable to identify cell subtypes as rare as 1% of the entire cell population.*

*We discussed the advantages of SnapATAC in identifying rare cell populations in the revised manuscript.*

*Graph 2. (a) TSNE diagram of the clustering result generated by APEC. Cluster 18, Sst╋Pvalb━ cells; cluster 22, Sst╋Pvalb╋ cells. (b) Expression of genes Sst and Pvalb, evaluated by APEC and imputed by MAGIC [32], same as Rongxin Fang et al used in their paper.*

5. In the computational method assessment paper, Chen H. et. al. explained that methods which preserve information at the peak/bin level performs better in distinguishing cell-types compared to TF-motif level or gene-centric level summarisation. Their interpretation for this was that the individual peaks are able to capture the complexity of one regulatory circuits more precisely. But APEC still outperforms the peak/bin based method (for eg. snapATAC) even though it is grouping peaks based on their synergistic signal patterns. What could be the possible reason behind this?

*Response: We think the reviewer raised up a very central question in single cell analysis and we would like to explain from the very beginning. First of all, every cell is different, one way or another. However, some cells are more similar than others, and thereby should be clustered together as cell subtypes. Secondly, signals we obtained from single cell sequencing technologies, especially on chromatin accessibility, come with a very high dropout/noise rate, resulting a very sparse fragment count matrix with a large loss of genomic information. It makes single cell clustering a great challenge. Thirdly, multiple algorithms, from TF-motif-centric (chromVAR), gene-centric (Cicero), peak-centric (LSI, cisTopic and APEC) to bin-centric (SnapATAC) were developed to address this challenge. We agree with Chen H.* [*el. al*](http://el.al)*. that the peak/bin-centric methods perform better in distinguishing cell types compared to the TF-motif or gene-centric approaches, since individual peaks/bins are able to capture the complexity of the regulatory circuits more precisely. Fourthly, the “gold standard” we use to evaluate the performance of a single cell clustering tool relies on our aprior knowledge of the cell types, which are usually identified by a limited number of genes or surface molecules that are biologically important. However, there are lots of accessible sites on the genome that have no known biological implications, but are contributing to the “mathematical” differences between the cells. The contradiction between the “biological” and “mathematical” assessment of the cells becomes more serious when the cells are very similar.*

*Taking together, in our humble opinion, a good scATAC-seq analysis algorithm needs to balance its ability to distinguish each single cell based on their incomplete genomic information and in the meantime to maintain the similarities of a group of cells that are “biologically” relevant without over emphasizing their “mathematical” difference. Take the HSC dataset as an example when cells are very similar, compare to the peak-centric methods like cisTopic and APEC, the bin-centric approach SnapATAC may have over emphasized the “mathematical” difference between the cells, resulting in several unidentifiable subgroups. (Fig. 1, Figure S2). However, when it comes to the mouse cell atlas dataset, SnapATAC works fast and well.*

*Unlike gene-centric methods such as Cicero, APEC is a peak-centric method (revised Figure S4). It groups peaks based on their synergistic signal patterns genome-wide, without any prior knowledge of the motifs or genomic loci of the peaks, which brings two major advantages: 1. The sparse cell-peak count matrix is converted to a dense cell-accesson count matrix (~600 accessons and a median ~40 peaks in each accesson), which maintains the distinctive characteristics of each single cell and in the meantime keeps the similarities between cells within a same subtype. 2. The covariation of the accessible sites across all cells reflects the spatial distance between the corresponding peaks, i.e. peaks in the same accesson are spatially close to each other (revised Figure S5), making APEC capable of maintaining the functional epigenetic signature of each cell. These are the main reasons why we believe APEC outperforms the rest of tools on the tested datasets.*

*Without question, SnapATAC has its unique advantages, especially in identifying rare cell types with extraordinary speed and scalability.*

6. When there would be minor clusters along with major clusters, for eg. with Cusanovich dataset, how APEC would perform? Although the authors have shown this in Figure 1(d) and S1, it was not obvious how the clustering was performed.

*Response: By default, APEC predicts the number of cell clusters by the Louvain algorithm. Based on this algorithm and the accesson count matrix obtained from the HSC dataset, APEC automatically separated CMP cells into 3 clusters (Fig. 1d and Figure S1). We observed that these 3 CMP clusters were enriched with different motifs (e.g. ERG, CTCF, and GATA), and were differentially distributed on the developmental trajectory (Fig. 3e).*

*To test the performance of APEC on identifying minor clusters along with major cluster, we took the reviewer’s suggestion and analyzed the Cusanovich’s dataset using APEC. We found that APEC automatically divided the entire dataset into 24 clusters. Most of the 24 cell clusters can be classified into the corresponding cell types in the original publication [2] (Graph 3a-c). Since Cusanovich et al. used a multi-layer clustering scheme and generated 40 cell types in their paper, several cell clusters in our results can be further distinguished. Take the cluster 13 as an example, we extracted these cells and re-clustered them using APEC. We found cluster 13 can be further separated into 12 sub-clusters (Graph 3d&e). Sub-cluster 1, 2, 7, 9, 10, 11 were loop of Henle cells, sub-cluster 4~6 were DCT/CD cells, and sub-cluster 0, 3, and 8 were proximal tubule, collecting duct, and distal convoluted tubule cells respectively (Graph 3f). In this way, APEC identifies minor clusters along with major clusters.*

*Graph 3. Comparison of clustering results from APEC versus published in Cusanovich et al. (a) Cell clusters obtained by APEC using the default parameters. (b) Cell types reported in Cusanovich et al. [2] (c) Percentage of cells in common of cell types defined in APEC versus in Cusanovich et al. (d) Sub-clusters generated by APEC for cells in cluster-13 in (a). (e) Cell types identified in Cusanovich et al. for cells in cluster-13 in (a). (f) Percentage of cells in common of sub-clusters identified in APEC versus in Cusanovich et al.*

7. How APEC would correct for batch effects if there is any and what would be its performance then?

*Response: The elimination of batch effect is another huge challenge in scATAC-seq analysis. The current version of APEC doesn’t support the function to correct the batch effect. For scATAC-seq dataset with strong batch effect, we recommend researchers to use other tools, such as SnapATAC, which was designed to merge different batches of datasets. We will try to embed batch merging algorithms, such as the canonical correlation analysis (CCA) and mutual nearest neighbor (MNN) methods, into the next version of APEC. We discussed the limitations of APEC in the revised manuscript.*

*References:*

*1	Cusanovich DA, Daza R, Adey A, Pliner HA, Christiansen L, Gunderson KL, Steemers FJ, Trapnell C, Shendure J: Multiplex single cell profiling of chromatin accessibility by combinatorial cellular indexing. Science 2015, 348:910-914.*

*2	Cusanovich DA, Hill AJ, Aghamirzaie D, Daza RM, Pliner HA, Berletch JB, Filippova GN, Huang XF, Christiansen L, DeWitt WS, et al: A Single-Cell Atlas of In Vivo Mammalian Chromatin Accessibility. Cell 2018, 174:1309-+.*

*3	Bravo González-Blas C, Minnoye L, Papasokrati D, Aibar S, Hulselmans G, Christiaens V, Davie K, Wouters J, Aerts S: <em>Cis-topic</em> modelling of single cell epigenomes. bioRxiv 2018.*

*4	Pliner HA, Packer JS, McFaline-Figueroa JL, Cusanovich DA, Daza RM, Aghamirzaie D, Srivatsan S, Qiu XJ, Jackson D, Minkina A, et al: Cicero Predicts cis-Regulatory DNA Interactions from Single-Cell Chromatin Accessibility Data. Molecular Cell 2018, 71:858-+.*

*5	Schep AN, Wu B, Buenrostro JD, Greenleaf WJ: chromVAR: inferring transcription-factor-associated accessibility from single-cell epigenomic data. Nat Methods 2017, 14:975-978.*

*6	Fang R, Preissl S, Hou X, Lucero J, Wang X, Motamedi A, Shiau AK, Mukamel EA, Zhang Y, Behrens MM, et al: Fast and Accurate Clustering of Single Cell Epigenomes Reveals <em>Cis</em>-Regulatory Elements in Rare Cell Types. bioRxiv 2019.*

*7	Splinter E, Heath H, Kooren J, Palstra RJ, Klous P, Grosveld F, Galjart N, de Laat W: CTCF mediates long-range chromatin looping and local histone modification in the beta-globin locus. Genes & Development 2006, 20:2349-2354.*

*8	Handoko L, Xu H, Li GL, Ngan CY, Chew E, Schnapp M, Lee CWH, Ye CP, Ping JLH, Mulawadi F, et al: CTCF-mediated functional chromatin interactome in pluripotent cells. Nature Genetics 2011, 43:630-U198.*

*9	Ong CT, Corces VG: CTCF: an architectural protein bridging genome topology and function. Nature Reviews Genetics 2014, 15:234-246.*

*10	Narendra V, Rocha PP, An DS, Raviram R, Skok JA, Mazzoni EO, Reinberg D: CTCF establishes discrete functional chromatin domains at the Hox clusters during differentiation. Science 2015, 347:1017-1021.*

*11	Tang ZH, Luo OJ, Li XW, Zheng MZ, Zhu JJ, Szalaj P, Trzaskoma P, Magalska A,* Wlodarczyk J, *Ruszczycki B, et al: CTCF-Mediated Human 3D Genome Architecture Reveals Chromatin Topology for Transcription. Cell 2015, 163:1611-1627.*

*12	Buenrostro JD, Corces MR, Lareau CA, Wu B, Schep AN, Aryee MJ, Majeti R, Chang HY, Greenleaf WJ: Integrated Single-Cell Analysis Maps the Continuous Regulatory Landscape of Human Hematopoietic Differentiation. Cell 2018, 173:1535-1548 e1516.*

*13	Cao JY, Cusanovich DA, Ramani V, Aghamirzaie D, Pliner HA, Hill AJ, Daza RM, McFaline-Figueroa JL, Packer JS, Christiansen L, et al: Joint profiling of chromatin accessibility and gene expression in thousands of single cells. Science 2018, 361:1380-1385.*

*14	Lake BB, Chen S, Sos BC, Fan J, Kaeser GE, Yung YC, Duong TE, Gao D, Chun J, Kharchenko PV, Zhang K: Integrative single-cell analysis of transcriptional and epigenetic states in the human adult brain. Nat Biotechnol 2018, 36:70-80.*

*15	Chen S, Lake BB, Zhang K: High-throughput sequencing of the transcriptome and chromatin accessibility in the same cell. Nature Biotechnology 2019, 37:1452-+.*

*16	Granja JM, Klemm S, McGinnis LM, Kathiria AS, Mezger A, Corces MR, Parks B, Gars E, Liedtke M, Zheng GXY, et al: Single-cell multiomic analysis identifies regulatory programs in mixed-phenotype acute leukemia. Nature Biotechnology 2019, 37:1458-+.*

*17	Chen HD, Lareau CA, Andreani T, Vinyard ME, Garcia SP, Clement K, Andrade-Navarro M, Buenrostro JD, Pinello L: Assessment of computational methods for the analysis of single-cell ATAC-seq data. Genome Biology 2019, 20.*

*18	Pliner HA, Packer JS, McFaline-Figueroa JL, Cusanovich DA, Daza RM, Aghamirzaie D, Srivatsan S, Qiu X, Jackson D, Minkina A, et al: Cicero Predicts cis-Regulatory DNA Interactions from Single-Cell Chromatin Accessibility Data. Mol Cell 2018, 71:858-871 e858.*

*19	Bravo Gonzalez-Blas C, Minnoye L, Papasokrati D, Aibar S, Hulselmans G, Christiaens V, Davie K, Wouters J, Aerts S: cisTopic: cis-regulatory topic modeling on single-cell ATAC-seq data. Nat Methods 2019, 16:397-400.*

*20	Saelens W, Cannoodt R, Todorov H, Saeys Y: A comparison of single-cell trajectory inference methods. Nature Biotechnology 2019, 37:547-+.*

*21	Stuart T, Butler A, Hoffman P, Hafemeister C, Papalexi E, Mauck WM, Hao YH, Stoeckius M, Smibert P, Satija R: Comprehensive Integration of Single-Cell Data. Cell 2019, 177:1888-+.*

*22	Welch JD, Kozareva V, Ferreira A, Vanderburg C, Martin C, Macosko EZ: Single-Cell Multi-omic Integration Compares and Contrasts Features of Brain Cell Identity. Cell 2019, 177:1873-+.*

*23	Doulatov S, Notta F, Eppert K, Nguyen LT, Ohashi PS, Dick JE: Revised map of the human progenitor hierarchy shows the origin of macrophages and dendritic cells in early lymphoid development. Nature Immunology 2010, 11:585-U552.*

*24	Karamitros D, Stoilova B, Aboukhalil Z, Hamey F, Reinisch A, Samitsch M, Quek L, Otto G, Repapi E, Doondeea J, et al: Single-cell analysis reveals the continuum of human lympho-myeloid progenitor cells. Nature Immunology 2018, 19:85-+.*

*25	Ciofani M, Zuniga-Pflucker JC: The thymus as an inductive site for T lymphopoiesis. Annu Rev Cell Dev Biol 2007, 23:463-493.*

*26	Park JE, Botting RA, Dominguez Conde C, Popescu DM, Lavaert M, Kunz DJ, Goh I, Stephenson E, Ragazzini R, Tuck E, et al: A cell atlas of human thymic development defines T cell repertoire formation. Science 2020, 367.*

*27	Buenrostro JD, Wu B, Litzenburger UM, Ruff D, Gonzales ML, Snyder MP, Chang HY, Greenleaf WJ: Single-cell chromatin accessibility reveals principles of regulatory variation. Nature 2015, 523:486-490.*

*28	Preissl S, Fang R, Huang H, Zhao Y, Raviram R, Gorkin DU, Zhang Y, Sos BC, Afzal V, Dickel DE, et al: Single-nucleus analysis of accessible chromatin in developing mouse forebrain reveals cell-type-specific transcriptional regulation. Nat Neurosci 2018, 21:432-439.*

*29	Cusanovich DA, Reddington JP, Garfield DA, Daza RM, Aghamirzaie D, Marco-Ferreres R, Pliner HA, Christiansen L, Qiu X, Steemers FJ, et al: The cis-regulatory dynamics of embryonic development at single-cell resolution. Nature 2018, 555:538-542.*

*30	Chen X, Litzenburger UM, Wei Y, Schep AN, LaGory EL, Choudhry H, Giaccia AJ, Greenleaf WJ, Chang HY: Joint single-cell DNA accessibility and protein epitope profiling reveals environmental regulation of epigenomic heterogeneity. Nat Commun 2018, 9:4590.*

*31	Huang M, Wang J, Torre E, Dueck H, Shaffer S, Bonasio R, Murray JI, Raj A, Li M, Zhang NR: SAVER: gene expression recovery for single-cell RNA sequencing. Nat Methods 2018, 15:539-542.*

*32	van Dijk D, Sharma R, Nainys J, Yim K, Kathail P, Carr AJ, Burdziak C, Moon KR, Chaffer CL, Pattabiraman D, et al: Recovering Gene Interactions from Single-Cell Data Using Data Diffusion. Cell 2018, 174:716-+.*

**Second round of review**

**Reviewer 1**

The revised manuscript have addressed my previous comments.

**Reviewer 2**

I thank the authors for addressing all the issues I raised in my earlier review. I am happy to accept the paper.
